# Functional fine-mapping of noncoding risk variants in amyotrophic lateral sclerosis utilizing convolutional neural network

**DOI:** 10.1038/s41598-020-69790-6

**Published:** 2020-07-30

**Authors:** Ali Yousefian-Jazi, Min Kyung Sung, Taeyeop Lee, Yoon-Ho Hong, Jung Kyoon Choi, Jinwook Choi

**Affiliations:** 1 Interdisciplinary Program, Bioengineering Major, Graduate School, Seoul National University, Seoul, 151-742 Republic of Korea; 2 MRC Laboratory of Molecular Biology, Francis Crick Avenue, Cambridge, CB2 0QH UK; 3 Graduate School of Medical Science and Engineering, KAIST, Daejeon, 34141 Republic of Korea; 4 Department of Neurology, Seoul Metropolitan Government Boramae Medical Center, Seoul National University College of Medicine, Neuroscience Research Institute, Seoul National University Medical Research Council, Seoul, Republic of Korea; 5 Department of Bio and Brain Engineering, KAIST, Daejeon, 34141 Republic of Korea; 6 Department of Biomedical Engineering, College of Medicine, Seoul National University, Seoul, 110-744 Republic of Korea

**Keywords:** Genome-wide association studies, Epigenomics, Machine learning, Amyotrophic lateral sclerosis

## Abstract

Recent large-scale genome-wide association studies have identified common genetic variations that may contribute to the risk of amyotrophic lateral sclerosis (ALS). However, pinpointing the risk variants in noncoding regions and underlying biological mechanisms remains a major challenge. Here, we constructed a convolutional neural network model with a large-scale GWAS meta-analysis dataset to unravel functional noncoding variants associated with ALS based on their epigenetic features. After filtering and prioritizing of candidates, we fine-mapped two new risk variants, rs2370964 and rs3093720, on chromosome 3 and 17, respectively. Further analysis revealed that these polymorphisms are associated with the expression level of CX3CR1 and TNFAIP1, and affect the transcription factor binding sites for CTCF, NFATc1 and NR3C1. Our results may provide new insights for ALS pathogenesis, and the proposed research methodology can be applied for other complex diseases as well.

## Introduction

Amyotrophic lateral sclerosis (ALS), also known as Lou Gehrig’s disease, is a late-onset neurodegenerative condition characterized by progressive wasting and weakness of limb, bulbar, and respiratory muscles, leading to death within 3–5 years from the onset of symptoms^1^. Although in most ALS patients the cause of the disease is unknown, at present a genetic cause is found in about 70% of familial ALS (FALS) patients and 10% of sporadic ALS (SALS) patients^2^. Genetic variants, including single-nucleotide polymorphisms (SNPs) and copy number variants, in the noncoding regions of the human genome can play an important role in human traits and complex diseases. Recently, genome-wide association studies (GWAS) have identified the common genetic variations that may contribute to the risk of ALS. To date, several GWAS have identified several risk loci for ALS. The most frequent genetic cause is a noncoding hexanucleotide repeat expansion in the C9orf72 gene. The other genes previously reported by GWAS are MOBP, UNC13A, TBK1, SCFD1, SARM1 and C21orf2 loci, all of which reached genome-wide significance^3^.

The efforts to decipher the biological consequences of noncoding variation face two major challenges. First, due to haplotype structure, GWAS tend to nominate large clusters of SNPs in linkage disequilibrium (LD), making it difficult to distinguish causal SNPs from neutral variants in the linkage. Second, even assuming the risk variants can be identified, interpretation is limited by incomplete knowledge of noncoding regulatory elements. Therefore, the researcher’s focus now shifts to accurate data interpretation and several approaches were proposed to predict functional noncoding variants. CADD^4^ and GWAVA^5^ are two recently published methods integrating functional genomic datasets to predict the deleteriousness of noncoding variants. In addition, the gkm-SVM^6^ and Trap^7^ were proposed to identify causative variants from sequence data. Recently, we proposed a scheme to combination of high-density genotyping and epigenomic data using a random forest model for discovering the autoimmune disease-specific noncoding risk variants^8^. Moreover, we proposed a post-GWAS analysis method using a convolutional neural network (CNN) trained on epigenetic features to find functional rare noncoding risk variants^9^. In this study, the CNN model was constructed with uncertain class labels on the epigenetic feature map extracted from the largest available GWAS data^3^ to predict functional noncoding variants associated with ALS.

## Results

### Overview of research methodology

**Figure 1:**
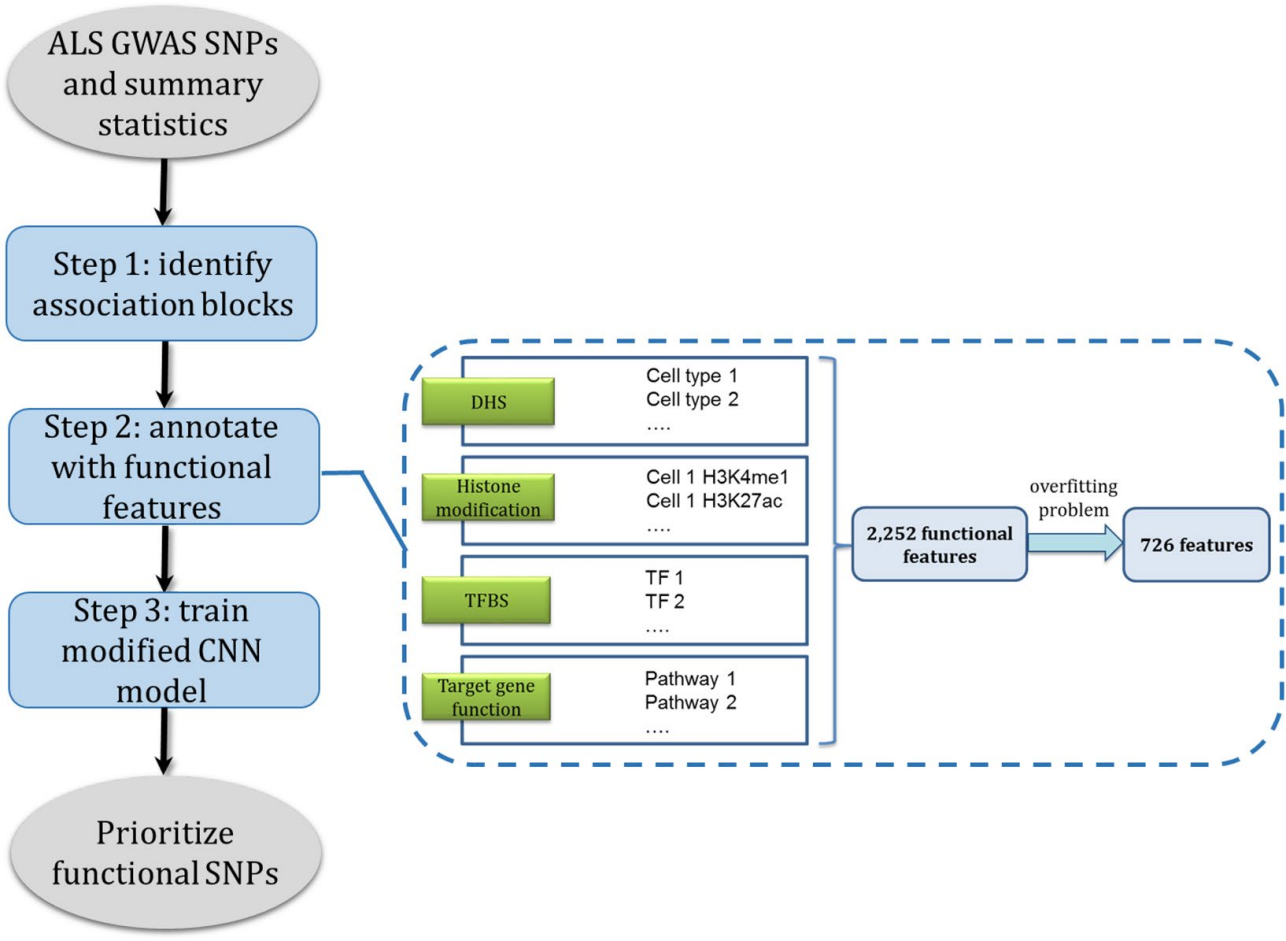
Outline of functional fine-mapping of ALS risk variants.

We used the genetic associations from a large-scale GWAS meta-analysis including 8,697,640 SNPs genotyped in 14,791 ALS patients and 26,898 healthy controls from 41 cohorts organized in 27 platform- and country-defined strata^3^. The research methodology in this study consists of three steps (Fig. 1): (1) define association blocks as follows. First, we discarded the SNPs with P > 5 × 10^–4^, then identified lead-SNPs which showed the strongest associations (the SNPs with the lowest *p *value) and 1 Mb apart from each other^10^. After that, we searched upstream and downstream regions flanking each lead SNP for the 30 most significant SNPs. Finally, we reached to 274 association blocks carrying the lead SNPs and their nonoverlapped neighboring SNPs. (2) Annotate each SNP with functional features from four different categories (“Methods” section), DHS mapping data, histone modifications, target gene functions, and transcription factor binding sites (TFBS). (3) Train the CNN model with uncertain labels (“Methods” section) on the extracted epigenetic feature map using a large number of hyperparameters and an autoencoder for pre-training. We split the input data to training, validation, and testing sets by chromosome^9,11^. Chromosomes 1–10 were used as the training set, and chromosomes 11–14 were used as the testing set that was used to report final performance levels. The best hyperparameter set was selected using Chromosomes 15–22 as the validation set. In the end, we prioritized the SNPs with a prediction score > 0.5 as the risk variant candidates.

### Biological characterization of noncoding risk variants

**Figure 2:**
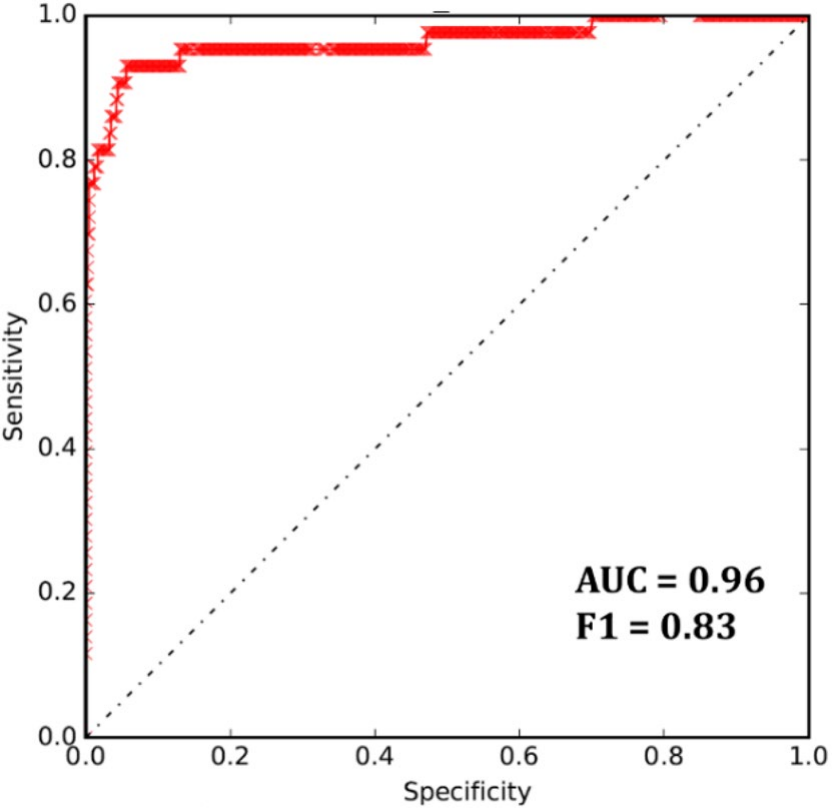
Model performance on the test set (Block-wised). ROC curve, AUC and F1 measured on the test set for the proposed CNN model using autoencoder pre-training process.

**Figure 3:**
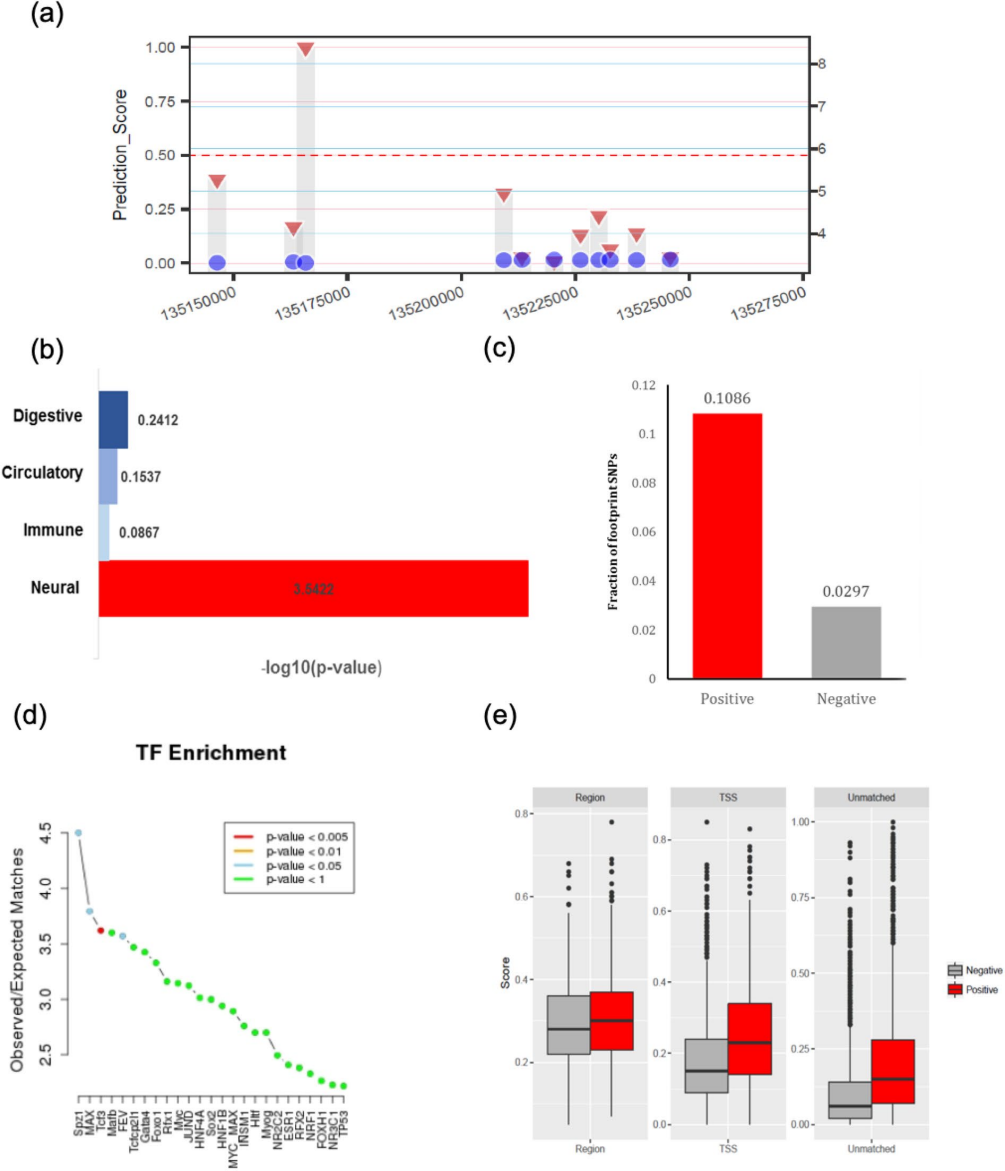
Analyzing prediction outcomes. (**a**) Comparison of the prediction scores (red triangles on the left y-axis) and association statistics (blue circles on the right y-axis) for individual SNPs in one association block. (**b**) Fraction of SNPs with the prediction score > 0.5 (positive) and < 0.5 (negative) located within TF binding sequences in > 40 cell types. (**c**) Overrepresentation of feature categories in the set of the significant Gini features as tested using the binomial distribution. (**d**) TF enrichment analysis for positively predicted SNPs (prediction_score > 0.5) using SNP2TFBS^14^. (**e**) Box-plot for GWAVA scores of three training sets for positive (red) and negative (gray) SNP groups.

The performance of our model was evaluated in terms of the area under the receiver operator characteristic curve (AUC) and F1 value (Fig. 2). In calculating the AUC, the true positive and true negative count to the association blocks with prediction_score > 0.5 or control blocks (“Methods” section) with prediction_score < 0.5. The validity of our results was tested in different ways. First, considering the risk variants are expected to have a certain level of statistical association with ALS, our results show 91 variants with the strongest statistical association (i.e., lead SNP) in 240 chromosomal blocks with at least one positive call (Fig. 3a). Moreover, a prominent role is expected for brain-related features when predicting risk variants associated with ALS. To test this, we employed the random forest classifier to assess the contribution of each feature to the prediction processes (“Methods” section). The neural features seemed to be more important in our model to characterize the ALS functional SNPs (Fig. 3b). On the other hand, noncoding causal variants may act through altering transcription factor (TF) binding. We used TF-contacting sequences identified by the nucleotide-resolution analysis of DHSs^12^. The fraction of sequences that were in physical contact with TFs was considerably higher for positive calls than negative calls (Fig. 3c). TCF3 is reported as one of the candidate causal master regulators of neurodegeneration in an in-vitro model of ALS^13^, and Fig. 3d shows TCF3 matching is significantly enriched in the positive SNPs group. In addition, we applied one of the state-of-the-art computational methods to predict the functional noncoding variants, GWAVA^5^. The higher GWAVA score means query SNP is more likely to be functional. In the same direction, the results showed higher GWAVA scores for positive than negative calls (Fig. 3e). Finally, the SNP feature map annotation indicates a significant difference in neural-related feature annotation between positive and negative groups (Fig. S.1).

### Filtering and prioritizing of risk variants and genes

**Figure 4:**
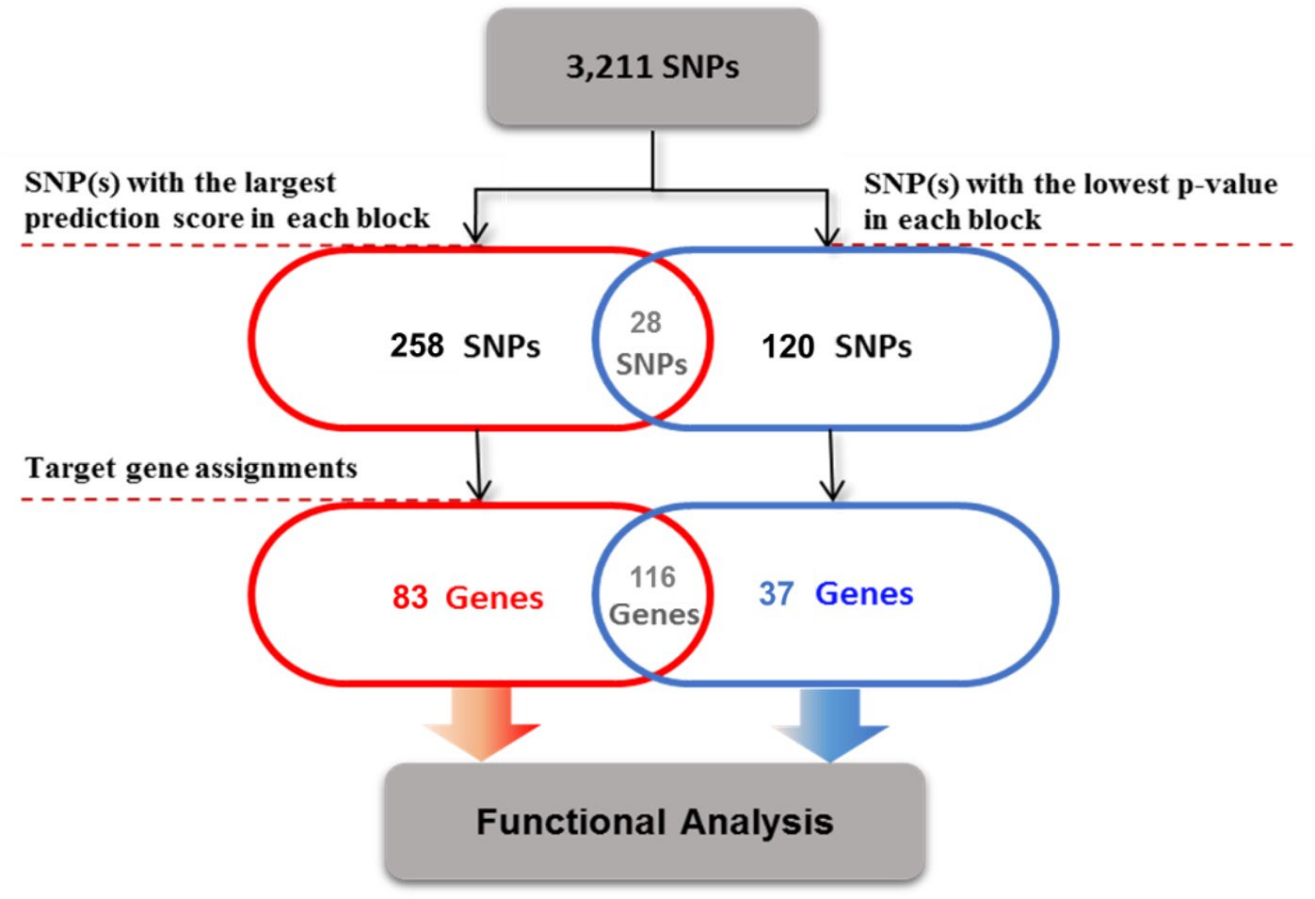
Gene set filtering pipeline. Workflow to reach a list of the most probable risk SNPs and genes.

**Figure 5:**
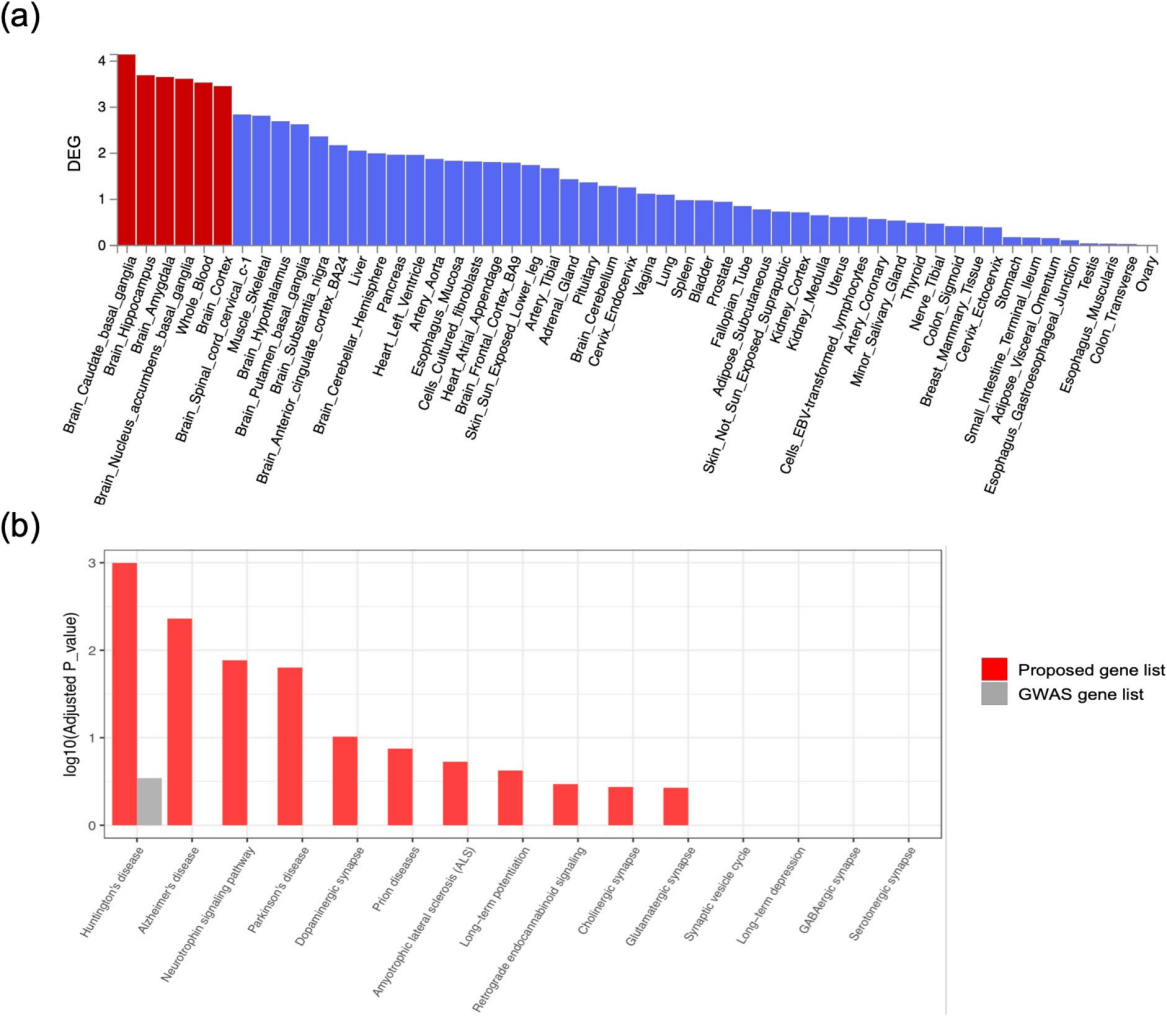
Characterization of the proposed potential risk genes and GWAS associated genes from the pipeline. (**a**) Enrichment of differentially expressed gene (DEG) for the proposed potential risk genes set in a certain tissue compared to all other tissue types. Red bars shows significant enrichment at Bonferroni corrected *p *value ≤ 0.05^17^. (**b**) Enrichment score for two groups of gene sets in the brain-related KEGG Pathway terms such as “nervous system” (“glutamatergic synapse”, “GABAergic synapse”, “cholinergic synapse”, “dopaminergic synapse”, “serotonergic synapse”, “long-term potentiation”, “long-term depression”, “retrograde endocannabinoid signaling”, “synaptic vesicle cycle”, and “neurotrophin signaling pathway”), and “neurodegenerative disease” (“Alzheimer’s disease”, “Parkinson’s disease”, “amyotrophic lateral sclerosis”, “Huntington’s disease”, and “prion diseases”).

In our results, 1,326 SNPs were predicted as putatively risk variants for ALS. In the process of interpreting our results in the search for risk SNPs, especially within noncoding regions, ruling-out false positives is of utmost priority. For this purpose, we defined a filtering pipeline (Fig. 4) to reach a list of the more probable risk SNPs and genes. Since the closest gene is typically not the target of transcriptional regulatory elements^15^, we considered 3 kb upstream of TSS as a promoter site and LASSO transcriptional enhancers in brain cell lines^16^ as an enhancer site for each gene for mapping a target gene to both proposed risk SNPs and GWAS associated SNPs. By applying this pipeline, we got to 286 SNPs and their related 199 genes as the more probable risk SNPs and genes for ALS (Table S.1). Then, we validate our results by performing several functional analysis on the 83 genes and 37 genes which are specifically categorized as potential risk genes and GWAS associated genes, respectively. For the first biological validation, we demonstrated that the proposed potential risk genes set are significantly expressed in the brain tissue (Fig. 5a), while the GWAS associated genes set are not enriched in the brain tissue (Fig. S.2). This analysis was performed by FUMA^17^, and identified tissue specificity of prioritized genes based on differentially expressed genes using GTEx v8 RNA-seq data for 54 tissue types^18^. For the second validation, we used the KEGG pathway^19^ terms belonging to related categories such as “nervous system”, and “neurodegenerative disease”. Figure 5b shows the enrichment of brain-related KEGG pathways for our proposed potential risk genes set.

### Functional assessment of noncoding risk variants and genes associated with ALS

Considering GWASs can only report large clusters of SNPs, or LD blocks, including not only causal variants but also many linked neutral SNPs, we wanted to look for variations more likely to be functional and genes neighboring GWAS tag-SNPs. Therefore, we considered SNPs which shared an association block with at least one significant GWAS associated SNP (*p *value < 5e−08). After mapping the target genes using promoter and enhancer sites, we compared the sets of genes associated with both groups of predicted risk SNPs and tag-SNPs (Table 1). As expected, some GWAS-associated genes were shared between both sets such as MOBP, C9orf72, SCFD1, SARM1, and UNC13A gene.

**Table 1:** Target genes assigned to proposed risk SNPs and GWAS tag-SNPs.

Proposed risk SNPs related genes	**MOBP**, *CX3CR1*, IFNK, **C9orf72**, MOB3B, **SCFD1**, *TNFAIP1*, **SARM1**, **UNC13A**
Tag-SNPs related genes	**MOBP**, IFNK, **C9orf72**, MOB3B, **LOC101927815**, **TBK1**, WASHC1, **SCFD1**, **SARM1**, SLC46A1, **UNC13A**, **C21orf2**

Bolded are the previously known genes for ALS and in italics are the proposed risk ALS genes.

**Figure 6:**
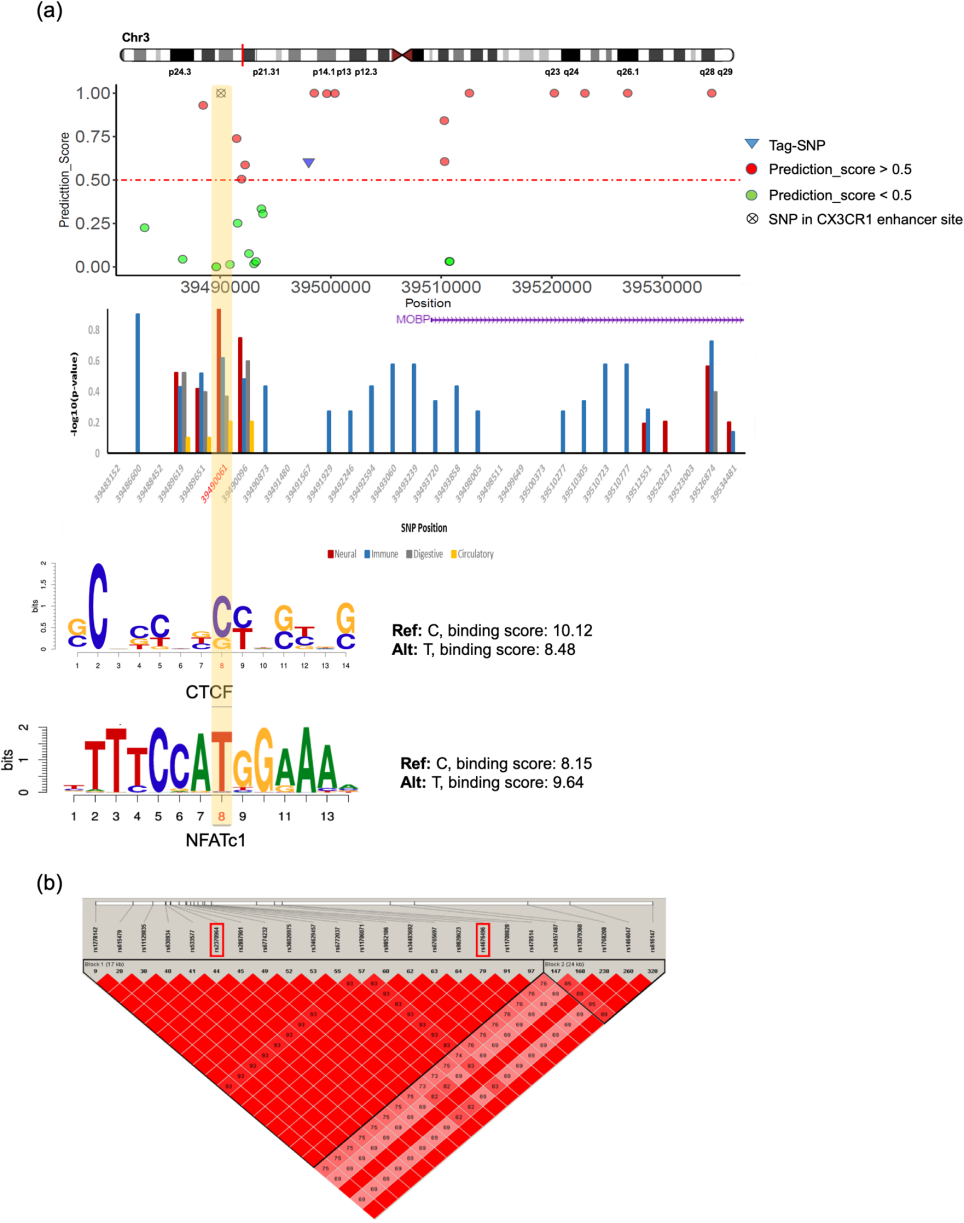
Functional analysis of the association block sharing MOBP gene. (**a**) The top shows prediction scores for all SNPs in an association block harboring a known ALS gene, MOBP on chromosome 3. Red and green circles represent associated SNPs with prediction_score > 0.5, prediction_score < 0.5, respectively; wheel cross represents SNP in CX3CR1 gene enhancer site. In the middle is an individual SNP feature map for all 30 SNPs inside of the block. The Y-axis is the negative logarithm of *p* value calculated based on a binomial test for multiple comparisons. The bottom is the CTCF binding site affinity for reference and alternative alleles. (**b**) Schematic locations of SNPs in the association block sharing MOBP gene along with LD blocks generated by Haploview.

There is no GWAS tag-SNP associated with the CX3CR1 (chemokine (C-X3-C motif) receptor 1) gene. Whereas, recently, it was reported that the V249I and T280M polymorphisms of the CX3CR1 gene are associated with the risk of ALS and modify phenotype in a large population-based series of ALS patients^20^. To explore the potential function of CX3CR1 in the brain, we explored CX3CR1 expression in different cell types of the central nervous system using the data from^21^. Figure S.3 also shows the CX3CR1 gene is highly expressed in microglia cells of human and mice brain^22^. Moreover, this gene has been proposed as a key mediator of neuron-microglia interactions that is upregulated under inflammatory conditions^23,24^. In our results, we first focused on the positively predicted SNP, rs2370964, in the enhancer site of the CX3CR1 gene. The association block close to the MOBP and CX3CR1 genes is shown in Fig. 6a, along with the SNPs feature map annotation plot. The variant with the strongest statistical association (chr3:39498005) was in the positive group, and the proposed SNP (chr3:39490061) annotated more in the neural-related features group as expected. According to the LD blocks shown in Fig. 6b for the SNPs in the interest association block, rs2370964 with the allele frequency of 49% is in the strong LD (D’ = 1, r^2^ = 0.98) with GWAS tag-SNP (rs4676496) and is considered as a putative risk SNP for ALS.

**Figure 7:**
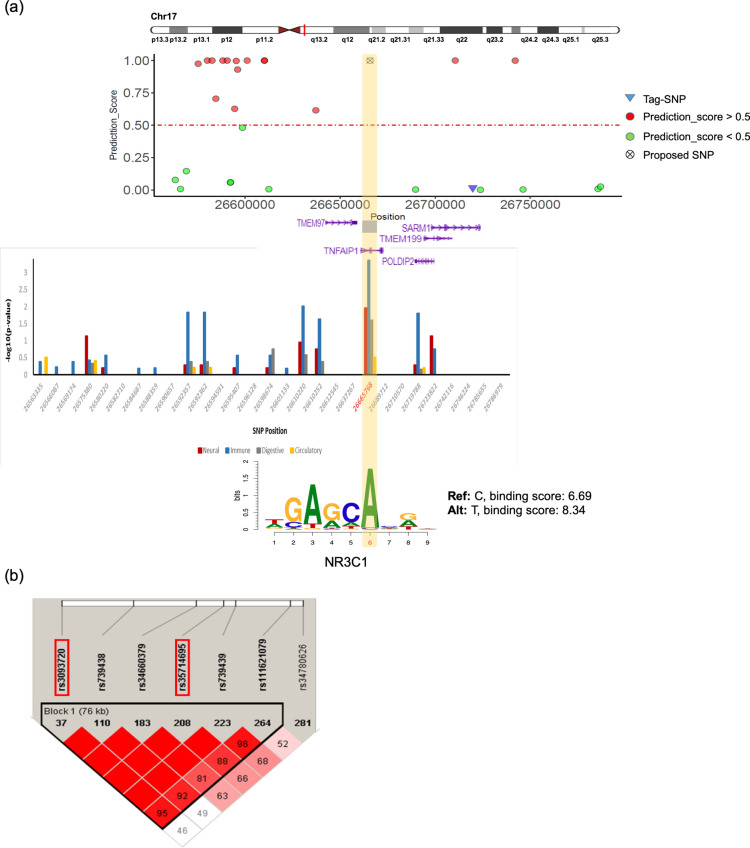
Functional analysis of the association block sharing SARM1 gene. (**a**) The top shows prediction scores for all SNPs in an association block harboring a known ALS gene, SARM1 on chromosome 17. Red and green circles represent associated SNPs with prediction_score > 0.5, prediction_score < 0.5, respectively; wheel cross represents SNP hit in the TNFAIP1 gene. In the middle is an individual SNP feature map for all 29 SNPs inside of the block. The Y-axis is the negative logarithm of *p *value calculated based on a binomial test for multiple comparisons. The bottom is CTCF binding site affinity for reference and alternative alleles. (**b**) Schematic locations of SNPs located close to SARM1 gene along with LD blocks generated by Haploview.

The second focus in our results is the intron variant, chr17:26665768, which hits tumor necrosis factor-induced protein 1 (TNFAIP1) gene close to SARM1 gene. The rs3093720 enriched more in immune and neural annotated features. While ALS is not primarily considered an autoimmune or immunodeficiency disease, mounting evidence suggests that immune/inflammatory abnormalities and non-neuronal cells play an important role in disease onset and progression^25^. Morello et al.^25^ distinguished the two sporadic ALS (SALS) subtypes, SALS1 and SALS2, each being associated with differentially expressed genes and pathways, and showed that TNFAIP1 is a neuroinflammatory gene differentially expressed in SALS2. This gene was predominantly up-regulated in the transgenic *Caenorhabditis elegans* Alzheimer's disease (AD) model and was also shown to have increased transcript levels in AD brains^26^. Furthermore, a strong LD (D’ = 1, r^2^ = 0.56) among g. 26665768 C > A with an allele frequency of 21%, and g. 26719788 G > A was identified in the European population (Fig. 7b).

**Figure 8:**
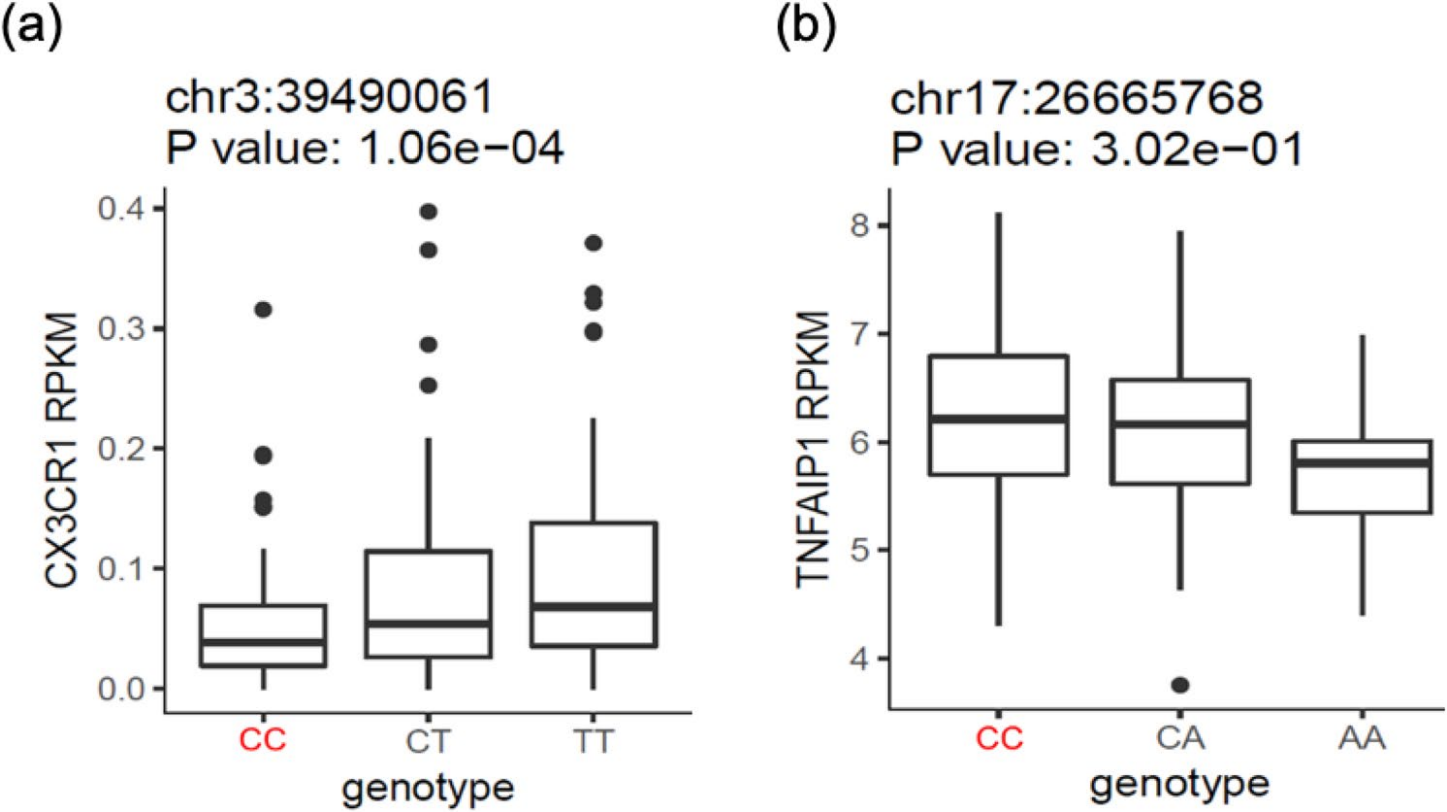
The eQTL analysis on lymphoblastoid cell lines. (**a**) The eQTL analysis for rs2370964 on CX3CR1 gene. (**b**) The eQTL analysis for rs3093720 on TNFAIP1 gene. Red is the risk-allele. RPKM: reads per kilobase per million.

Considering the two identified SNPs are located in a non-coding region, it is likely that these variants exert their effects on ALS through affecting gene expression. In this study, we used expression quantitative trait loci (eQTL) which is one of the most prominent methods for discovery of genetic variants that explain variation in gene expression levels. eQTL analysis on RNA sequencing data from lymphoblastoid cell lines of 465 individuals from the 1,000 Genomes Project^27^ shows the reference and risk allele, *C*, is responsible for the reduction of CX3CR1 expression levels (Fig. 8a). Deletion of CX3CR1 in a transgenic model of ALS mice was shown to exacerbate neuronal cell loss, suggesting that CX3CL1/CX3CR1 signaling limits microglial toxicity in ALS^24^. On the other hand, Fig. 8b shows the TNFAIP1 expression level decrease by alternative allele, A, by the SNP of interest in lymphoblastoid cell lines. TNFAIP1 was originally identified as a gene whose expression can be induced by tumor necrosis factor alpha (TNFα) in umbilical vein endothelial cells^28^. Liu et al.^29^ demonstrated that TNFAIP1 can be induced by Aβ_25–35_, and overexpression of TNFAIP1 promotes Aβ_25–35_-induced neurotoxicity, whereas knock-down of TNFAIP1 blocks Aβ_25–35_-induced neurotoxicity. These changes in gene transcription can result from changes in the TFBS motif. The rs2370964 polymorphism disrupts the binding sites for CTCF which is a DNA-binding protein that organizes nuclear chromatin topology. Mutations in CTCF cause intellectual disability and autistic features in humans, and McGill et al.^30^ found that CTCF depletion leads to overexpression of inflammation-related genes and microglial dysfunction. Moreover, Nagamoto-Combs et al. demonstrated that NFAT plays a role in regulating proinflammatory responses in cultured murine microglia, the resident immune cells of the central nervous system^31^. According to our results, this SNP also creates a new TFBS for the NFATc1 isoform. In the case of rs3093720, this SNP mostly annotated in immune and neural features groups, and effects the binding site of NR3C1 (Fig. 7a), a glucocorticoid receptor associated with elevated stress signaling in neurodegeneration^32^.

## Conclusion

Recent large-scale GWAS have identified multiple risk variants that show strong association with ALS. But, some of the rare variants might be missed in GWAS, fine- mapping and imputation statistical procedures^33^. Rare variants with greater effect sizes might confer highly deleterious effects on development or progression of ALS^34,35^, and thus, it is crucial to include them in the subsequent post-GWAS analysis. A number of methods have been developed for inferring noncoding risk variants using different functional data and computational methodologies. To our knowledge, the methodology proposed by Lee et al. is the most accurate and recent post-GWAS model for finding noncoding rare risk variants^9^, although we developed the CNN model by considering the concept with lack of a gold standard for labeling the association blocks. For the first time, we proposed the CNN model with uncertain class labels, and applied them in an attempt to predict noncoding risk variants on the basis of their functional features. Of importance, since our functional prediction method, only use the position of the associated SNPs and trained on the common patterns between annotated functional features shared by putative risk variants scattered among multiple associated loci, it is applicable to rare variants, and is able to single out one statistically indistinguishable variant from the GWAS variants (Fig. 3a). However, it may be possible that some false-positive variants are included, and a filtering pipeline was added to reach candidates functional risk SNPs for ALS. Our method led to a discovery of two putative ALS genes, CX3CR1 and TNFAIP1, and corresponding noncoding SNPs.

Several criteria need to be met for an SNP to be considered a causal variant in a disease such as ALS. Notably, the SNP should have an impact, probably small, on molecular or cellular systems of neural and/or related cells and/or tissue(s). It can also be the case that an SNP localized in a noncoding region is likely to affect the expression of one or several genes through different molecular mechanisms^36^. The eQTL analysis showed SNPs rs2370964 and rs3093720 may confer the risk of ALS through affecting CX3CR1 and TNFAIP1 expression (Fig. 8). These changes in gene transcription can result from changes in the TFBS motifs.

Both rs2370964 and rs3093720 are in the strong LD with GWAS tag-SNPs, and it provided additional evidence that supports rs2523593 being an SNP that confers a prominent risk for ALS. Our integrative analysis and gene expression results provide convergent lines of evidence that support the potential involvement of CX3CR1 and TNFAIP1 in ALS.

In this study, we get closer to clearly defining the risk variants and confidently declaring the genes as those implicated as causal variants in ALS; however, more work is needed to investigate the exact role of the proposed genes in the pathogenesis of ALS. Finally, further experimental strategies are necessary in order to effectively detect the potentially minuscule impact of two functional SNPs on putative risk genes.

## Methods

### Feature set annotation

Each SNP was annotated with 2,252 functional features from four different categories including: (1) DHS mapping data in 349 different samples covering 124 distinct cell types^37,38^, (2) 606 histone modification profiles in 127 human tissues or cell lines^38^, (3) 301 pathways from the KEGG database^19^ for function of target genes, and (4) transcription factor binding sites computed using FIMO^39^ at the *p *value threshold of 10^–4^ for 996 transcription factors from TRANSFAC^40^ and JASPAR^41^ databases.

We constructed a binary input matrix such as assigning 1 for each feature associated with the SNPs of interest and 0 otherwise. Because of dealing with the overfitting problem, the features that were not mapped to any SNPs in > 95% of the association blocks were excluded and the resulting number of surviving features was 726. Finally, we reached an input matrix of 274 association blocks including at most 30 SNPs in each block with 726 functional features for each SNP.

### CNN model with uncertain labeling

In real applications of machine learning problems, it is often the case that we cannot exactly obtain the true labels. In our problem, since we did not have a gold standard for labeling the association blocks, we modified the original CNN model used in^9^ based on the uncertain labeling concept in classification problems^42–44^. Our CNN model was constructed based on two convolution layers. The first layer applied a rectified linear unit (ReLU) and acts as a local feature extractor at the individual SNP level. The size of input matrix for this layer is *M* × *N* which *M* is the number of functional features that survived from the filtering step and *N* is the total number of candidate SNPs in each block. We applied 50 one-dimensional filters with a length of 726 (survived functional features) with a moving window of step size 1. In this way, 50 types of pattern detectors were used for each SNP without considering the effect of neighboring SNPs. After convolving the input matrix, and adding a bias vector, we applied ReLU to reach an output matrix *K* × *N* which *K* is the number of filters used in our model. The consequence matrix corresponds to per-SNP scores measuring how well the features of each SNP match the patterns of the shared weights. In the second convolutional layer, only one tunable weight vector was used to linearly combine the 50 patterns for high-level feature scoring of each SNP and sigmoid function scaled the results to the 0–1 range. The output from this layer can be considered as the prediction score of each SNP and the value close to 1 indicate that certain common regulatory patterns are embedded in the features of the given SNPs. Finally, max-pooling was applied to find the per-block score of the SNP whose features best match the common patterns shared by different blocks. More information can be found in^9^.

In the original model, all the association blocks (true cases) which are assumed to carry at least one causal SNP and control blocks (false cases) which are constructed by shuffling the regulatory features of SNPs in the true cases were labeled 1 and 0 respectively. Because of the lack of any gold standard for labeling the causal association blocks, it is not fair to simply assign either association or control blocks labels. Therefore, we used an extra weight for each block labels which show the certainty about labeling based on meta-analysis *p* value as follows:

$$W = \left\{ {\begin{array}{*{20}l} {10} \hfill & {if\quad minimum\;of\;p - value\;for\;a\;block < 5e - 08} \hfill \\ 8 \hfill & {if\quad 5e - 08 < minimum\;of\;p - value\;for\;a\;block < 5e - 07} \hfill \\ 6 \hfill & {if\quad 5e - 07 < minimum\;of\;p - value\;for\;a\;block < 5e - 06} \hfill \\ 4 \hfill & {if\quad 5e - 06 < minimum\;of\;p - value\;for\;a\;block < 5e - 05} \hfill \\ 2 \hfill & {if\quad 5e - 05 < minimum\;of\;p - value\;for\;a\;block < 5e - 04} \hfill \\ 2 \hfill & {false\;cases} \hfill \\ \end{array} } \right.$$

We generated false cases that are ten times the true cases. The loss function was composed of parameters (θ) including the weight vectors and biases in the first and second convolutional layers which were updated by the standard backpropagation algorithm with momentum. We also trained the model parameters to minimize a loss function defined as follows^9^:

$$LOSS = NLL + \lambda_{1} w_{1} + \lambda_{2} w_{2}$$

where *NLL* stands for the mean of negative log likelihood, and $\lambda_{1} w_{1} + \lambda_{2} w_{2}$ represents the regularization term of the elastic net that is used to control overfitting. The *NLL(θ)* of the loss function is given as

$$NLL\left( \theta \right) = - \frac{1}{B}\mathop \sum \limits_{m = 1}^{B} W^{m} (Y^{m} logf\left( \theta \right)^{m} + (1 - Y^{m} ){\log}\left( {1 - f\left( \theta \right)^{m} } \right))$$

where *Y* can be either 1 or 0 for true cases or false cases, respectively, and *f(θ)*^*m*^ is an output for the *m*^*th*^ GWAS block in a mini-batch of size *B* (B = 100). To allow the model to learn more robust features, we used the denoising autoencoder to pre-train the filters. Autoencoder is an unsupervised learning function which can be considered for fine-tuning by assigning an optimal starting point. In our model, autoencoder takes vectors of *M* functional features of each SNP as input, then was trained to reconstruct the input from a stochastically corrupted version. The stopping criteria and hyperparameters selection in this model can be found in^9^.

###  Feature importance analysis

One of the major criticisms of CNN models is their being black boxes, since no satisfactory explanation of the weights learned by CNN has been used for the assessment of feature importance. In this study, this drawback was tackled by employing random forest (RF) as a supervised ensemble learning method that operates by constructing several randomized decision trees. RF was trained on the labeled SNPs as positive (prediction_score > 0.5) or negative (prediction_score < 0.5) according to CNN results. 100 decision trees constituted the RF and 10 features were randomly sampled at each split. The Gini importance score was calculated to evaluate the relative importance of each feature. First, the response variable was randomly permutated 1,000 times, then feature importance from the permutated data was compared with the original importance levels. Finally, *p* values were estimated as the number of cases where permutated feature importance exceeded real importance using the hypergeometric distribution.

$$P\left( {X = k} \right) = \frac{{\left( {\begin{array}{*{20}c} K \\ k \\ \end{array} } \right)\left( {\begin{array}{*{20}c} {N - K} \\ {n - k} \\ \end{array} } \right)}}{{\left( {\begin{array}{*{20}c} N \\ n \\ \end{array} } \right)}}$$

where N is the total number of features, K is the number of all significant features (*p* value < 0.05), n and k are the number of features and the number of significant features in each category (Neural, Immune, Digestive and Circulatory) (Table S.2). We did not consider repressive histone marks, H3K9me3 and H3K27me3, because they are not specifically mapped to individual SNPs. We implemented RF by using R packages, randomForest and rfPermute^45^.

### Identification of linkage disequilibrium blocks

The structure of LD in the region was determined using Haploview^46^. We used the pedigree data of the specific chromosome region from the European population 1,000 Genomes project (phase 3 release) as an input for Haploview to identify SNPs in the same LD. Haplotype blocks were defined based on D’ estimates using the Solid Spine of the LD option.

## Supplementary information

Supplementary Figures.

Supplementary Table S.1.

Supplementary Table S.2.
