# Supplementary material for: Functional fine-mapping of noncoding risk variants in amyotrophic lateral sclerosis utilizing convolutional neural network
Source: Sci Rep. 2020 Jul 30;10:12872. doi: 10.1038/s41598-020-69790-6 (PMC7393092; doi:10.1038/s41598-020-69790-6)
Supplement: Supplementary file 1 — Supplementary Figures. [file 41598_2020_69790_MOESM1_ESM.docx]

**Functional fine-mapping of noncoding risk variants in amyotrophic lateral sclerosis utilizing convolutional neural network**

**Ali Yousefian-Jazi^1^, Min Kyung Sung^2^, Taeyeop Lee^3^, Yoon-Ho Hong^4^, Jung Kyoon Choi^5*^, Jinwook Choi^6*^**


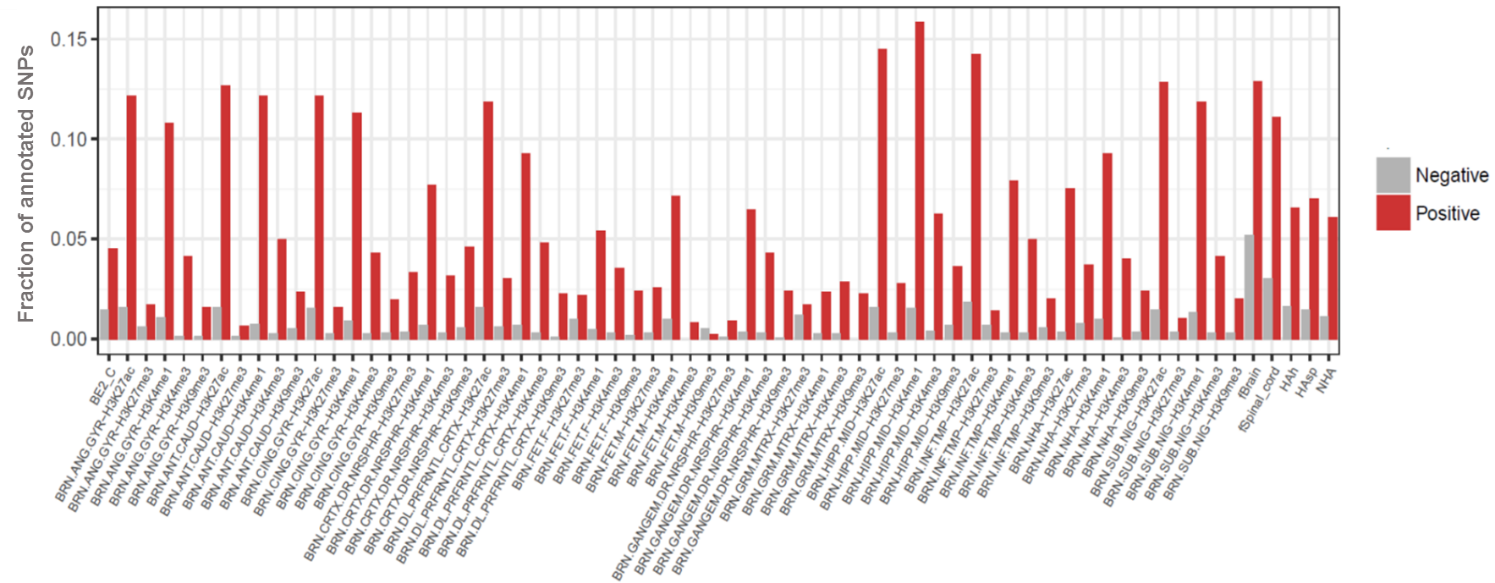


**Fig. S. 1. SNP annotation neural related features for positive and negative groups.**

Fraction of SNPs with the prediction score > 0.5 (positive) and < 0.5 (negative) annotated in each individual epigenetic feature in neural tissues.

**Fig. S. 2. Enrichment of differentially expressed gene (DEG) for the GWAS association genes set in a certain tissue compared to all other tissue types.** Red bars shows significant enrichment at Bonferroni corrected P-value ≤ 0.05

(a)


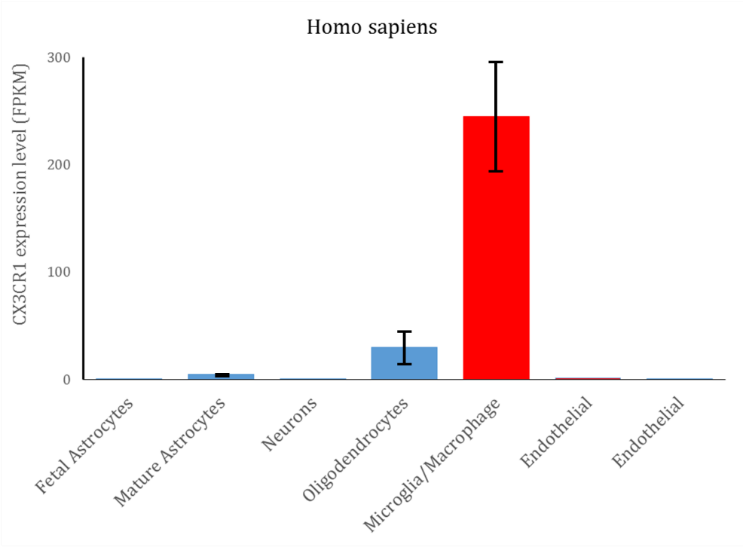


(b)


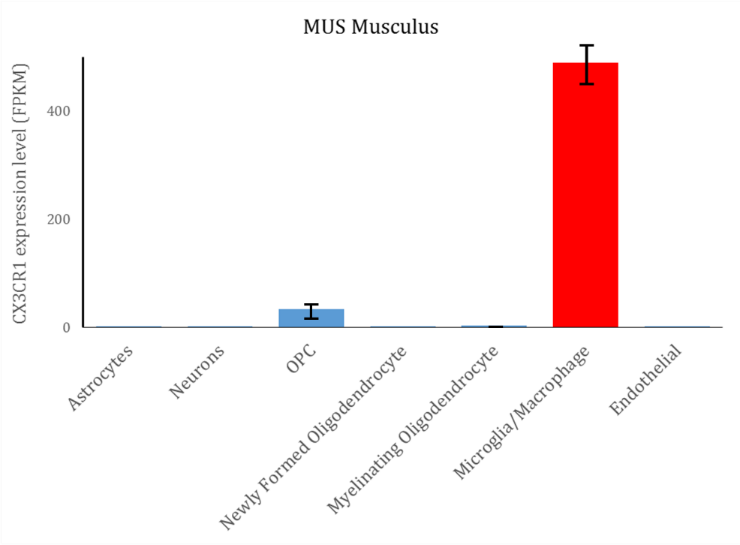


**Fig. S. 3. Expression of Cx3CR1 in different cell types of human and mouse brain.**

CX3CR1 gene is highly expressed in microglia cells of (a) human and (b) mouse brain. FPKM: fragments per kilobase of transcript per million.
